# Supplementary material for: Dissecting the basis for differential substrate specificity of ADAR1 and ADAR2
Source: Nat Commun. 2023 Dec 11;14:8212. doi: 10.1038/s41467-023-43633-0 (PMC10713624; doi:10.1038/s41467-023-43633-0)
Supplement: Supplementary file 1 — Supplementary Information [file 41467_2023_43633_MOESM1_ESM.pdf]

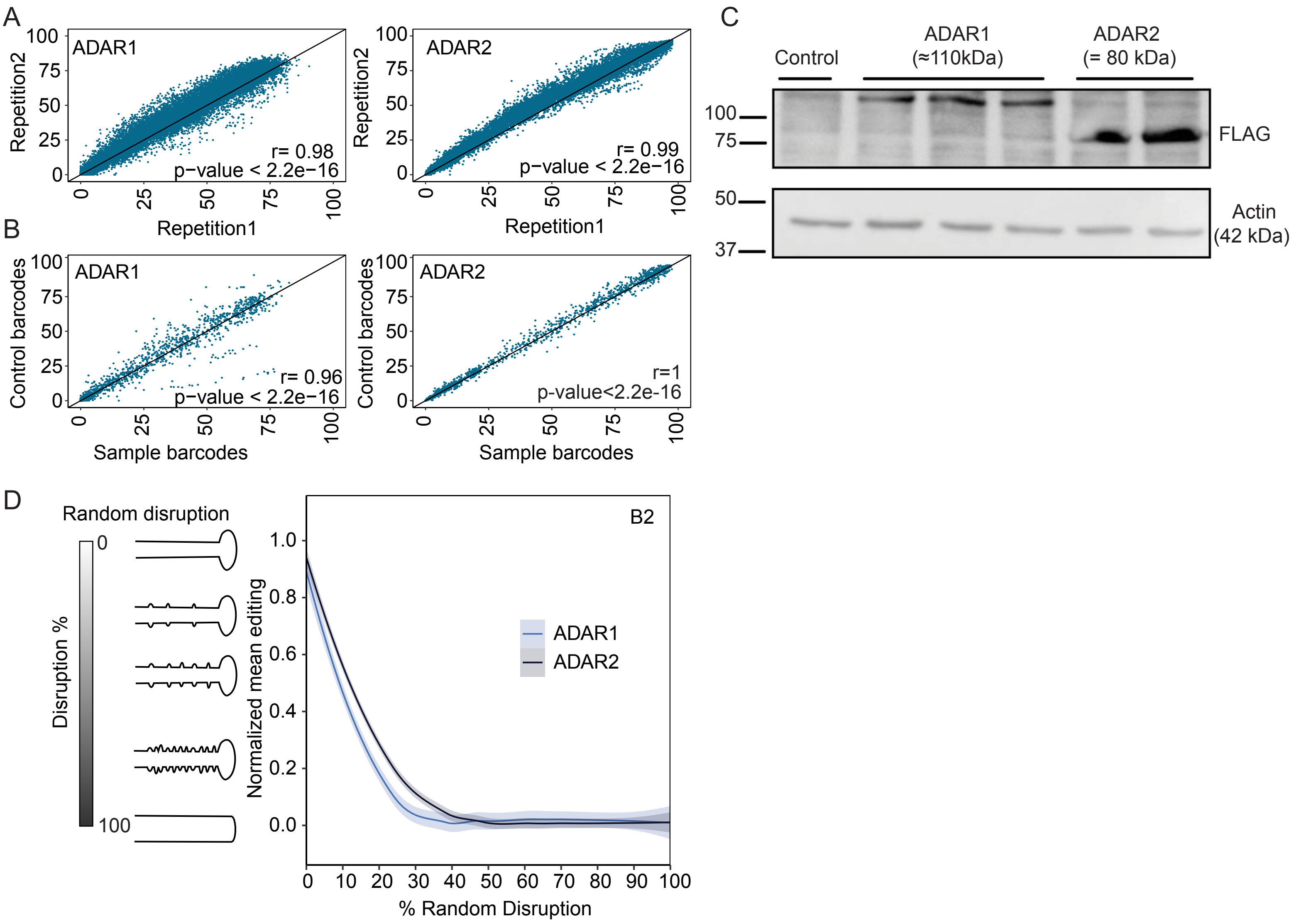

Figure S1. Systematic screening of ADAR2 synthetic substrates in ADAR1 knockout HEK-293T cell lines.

A) Correlation of A-to-I levels among technical replicates in cells overexpressing either ADAR1 or ADAR2. Each dot depicts the editing percentage of an adenosine in each construct of the mNG oligo library. The Pearson correlation coefficient and p-values are shown. B) Correlation of editing levels in mNG constructs that differ in the barcode sequences. The Pearson correlation coefficient and p-values are shown. C) Western blot of cell lysates from ADAR1-knockout cells overexpressing FLAG-tagged ADAR1 and ADAR2. The 80 and 110 kDa bands correspond to ADAR2 and ADAR1, respectively. Total RIPA cell lysates were prepared from ADAR1-knockout HEK 293T cells transfected 48h with respective ADAR-pcDNA3.1(-) overexpression plasmids and analyzed by Western blotting with antibodies specific for anti-mouse FLAG, anti-goat Actin HRP and anti-mouse HRP. D) Min-max normalized mean editing percentage in the series of B2 constructs containing random disruptions of double-strandedness in 5% increases.

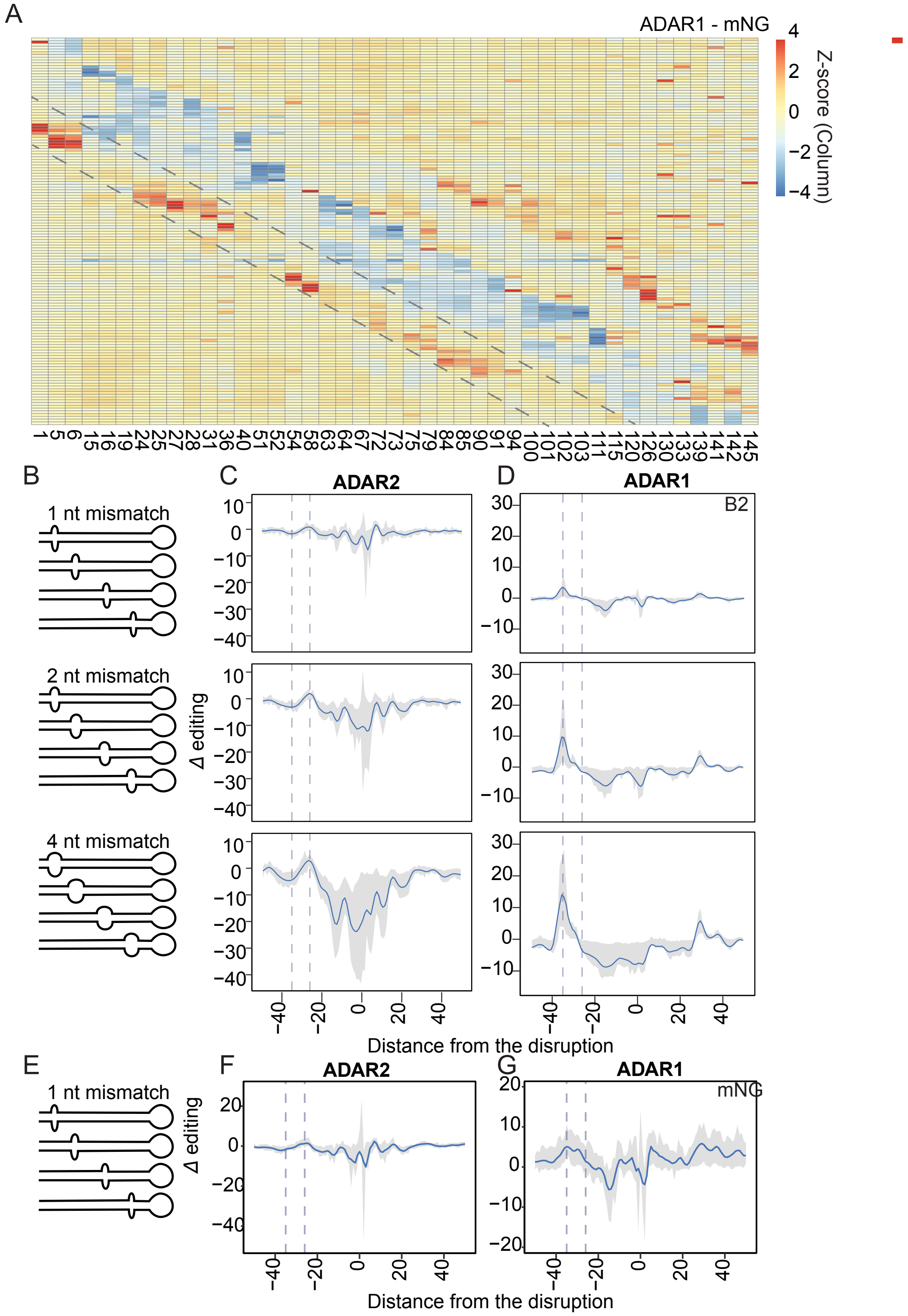

Figure S2. ADAR1- and ADAR2-mediated editing is induced at a constant interval of 26 bp upstream from structural disruptions. A) Heatmap of a 3-nucleotide mismatch running from 5' to 3' throughout the double-stranded RNA. Each row represents a construct structurally disrupted at a specific position while each column represents an adenosine position. Delta (*Δ*) editing is color-coded after scaling by columns using Z-score transformation (mNG series). The parallel dashed lines highlight the ADAR1-mediated editing increase at fixed distance upstream from the 3-nucleotide mismatch. B) Graphical scheme of subsets of B2 constructs carrying 1, 2 or 4 bp mismatches along the stem structures.C) ADAR2-mediated editing offset based on subsets of 1-, 2- and 4- nt mismatch running throughout the B2 sequences. Mismatches differentially located in each construct get centered at 0 on the x-axis. The *Δ* of the editing level on the y-axis represents the change of editing level of an adenosine, normalized to the perfect double-stranded construct. Fitted curves depict LOESS fit of *Δ* editing with a span of 0.05. The shaded region spans the 25th Percentile and 75th percentile values of *Δ* editing per distance. Only adenosine positions, which have greater than 1% in editing, on the perfect double-stranded construct were included in the analysis. Vertical dashed lines are placed at -26 and -35. D) ADAR1-mediated editing offset based on subsets of 1-,2 and 4- nucleotide mismatch running throughout the B2 sequences. Data is shown as in the figure S2C. E) Graphical scheme of subsets of mNG constructs carrying 1 bp mismatch along the stem structures. F) ADAR2-mediated editing offset based on the subset of 1-nucleotide mismatch running throughout the mNG sequences. Data is shown as in the figure S2C. G) ADAR1-mediated editing offset based on the subset of 1-nucleotide mismatch running throughout the mNG sequences. Data is shown as in the figure S2C.

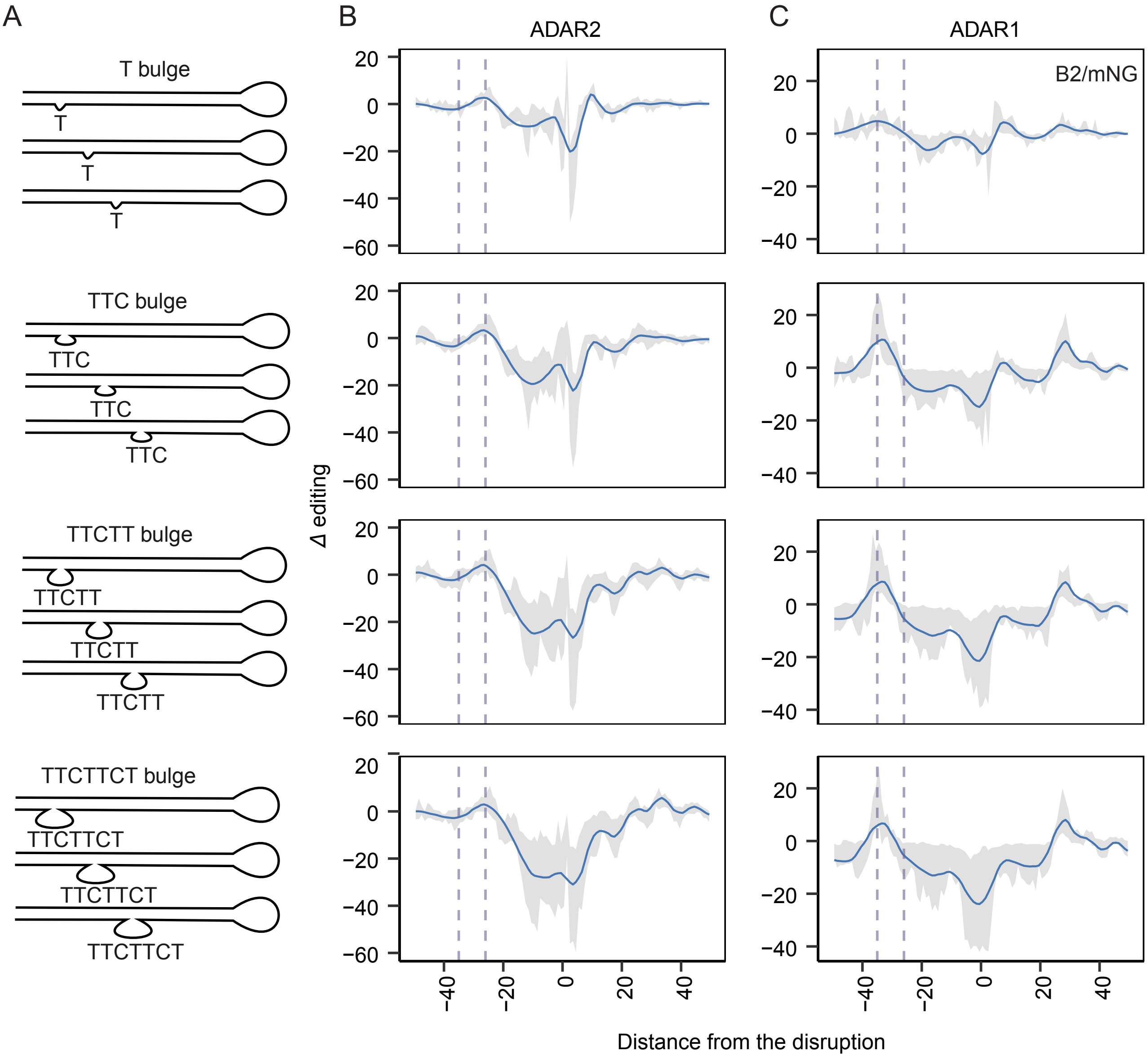

Figure S3. ADAR1- and ADAR2-mediated editing is induced at a constant interval of 35 bp and 26 bp, respectively, upstream from pyrimidine-rich bulges. A) Graphical scheme of subsets of B2 and mNG constructs carrying T, TTC, TTCTT and TTCTTCT bulges along the stem structures. B) ADAR2-mediated editing offsets based on subsets of T, TTC, TTCTT and TTCTTCT bulge running throughout the B2 and mNG sequences. Bulges differentially located in each construct get centered at 0 on the x-axis. The *Δ* editing level on the y-axis represents the change of editing level of an adenosine, normalized to the perfect double-stranded construct. Fitted curves depict LOESS fit of *Δ* editing with a span of 0.11. The shaded region spans the 25th-75th percentile values of *Δ* editing per distance. Vertical dashed lines are placed at -26 and -35. C) ADAR1-mediated editing offsets based on subsets of T, TTC, TTCTT and TTCTTCT bulge running throughout the B2 sequences. Data is shown as in the figure S3B**.**

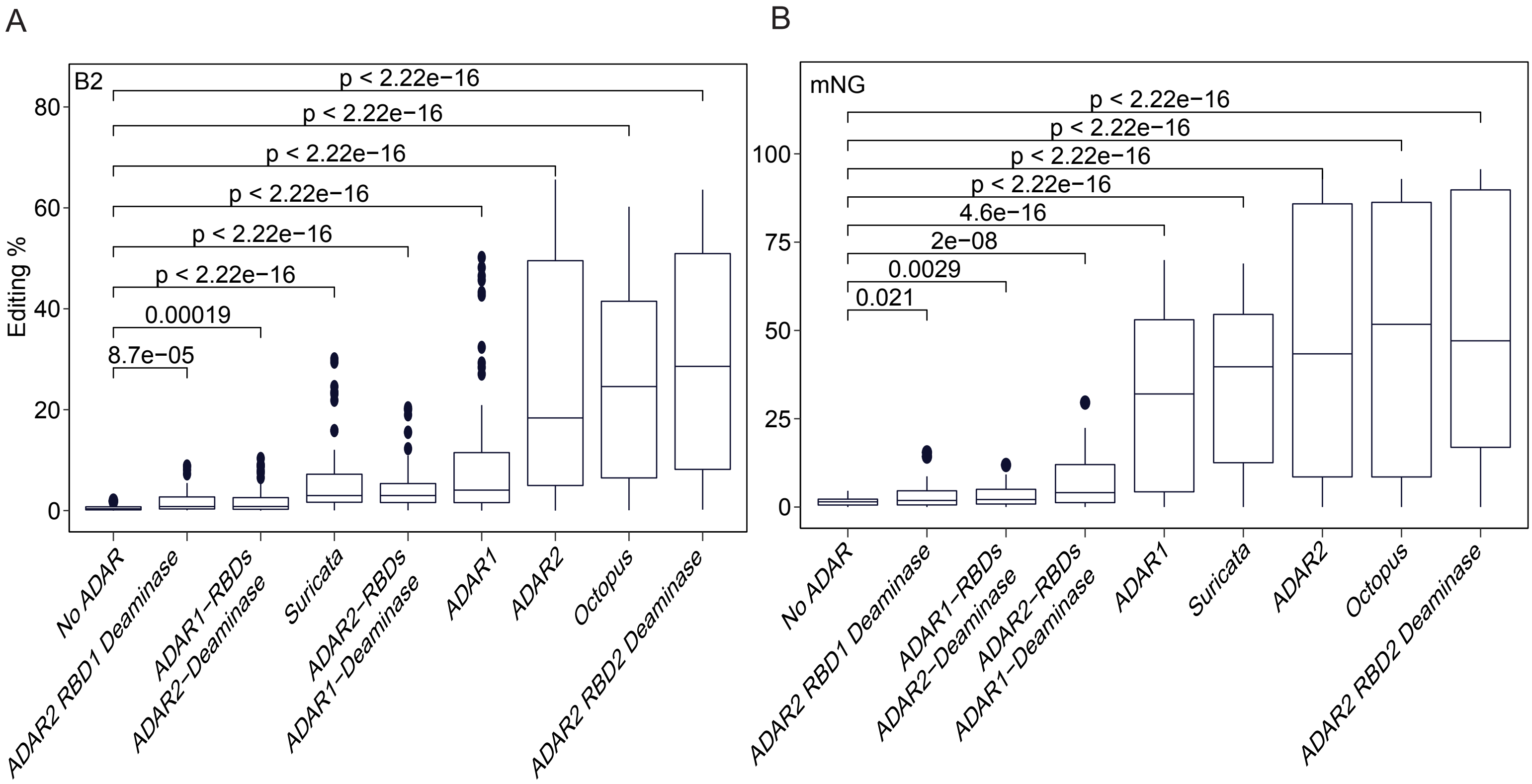

Figure S4. Editing levels of synthetic substrates among different ADARs in ADAR1-knockout HEK293T cell lines. A) Distribution of fraction of edits per position on B2 perfect double-stranded reporter. Data from two replicates are combined and the pairwise comparisons were evaluated using Wilcoxon-test. The corresponding p-values are shown on the top of the barplots. Data is visualized via box-and-whisker plots, with the central line denoting the median, box edges representing the interquartile range (from the 25th to the 75th percentile), whiskers indicating the 1.5 times interquartile range, and outliers are included. B) Distribution of fraction of edits per position on mNG perfect double-stranded reporter. Data is shown as in Figure S4A.

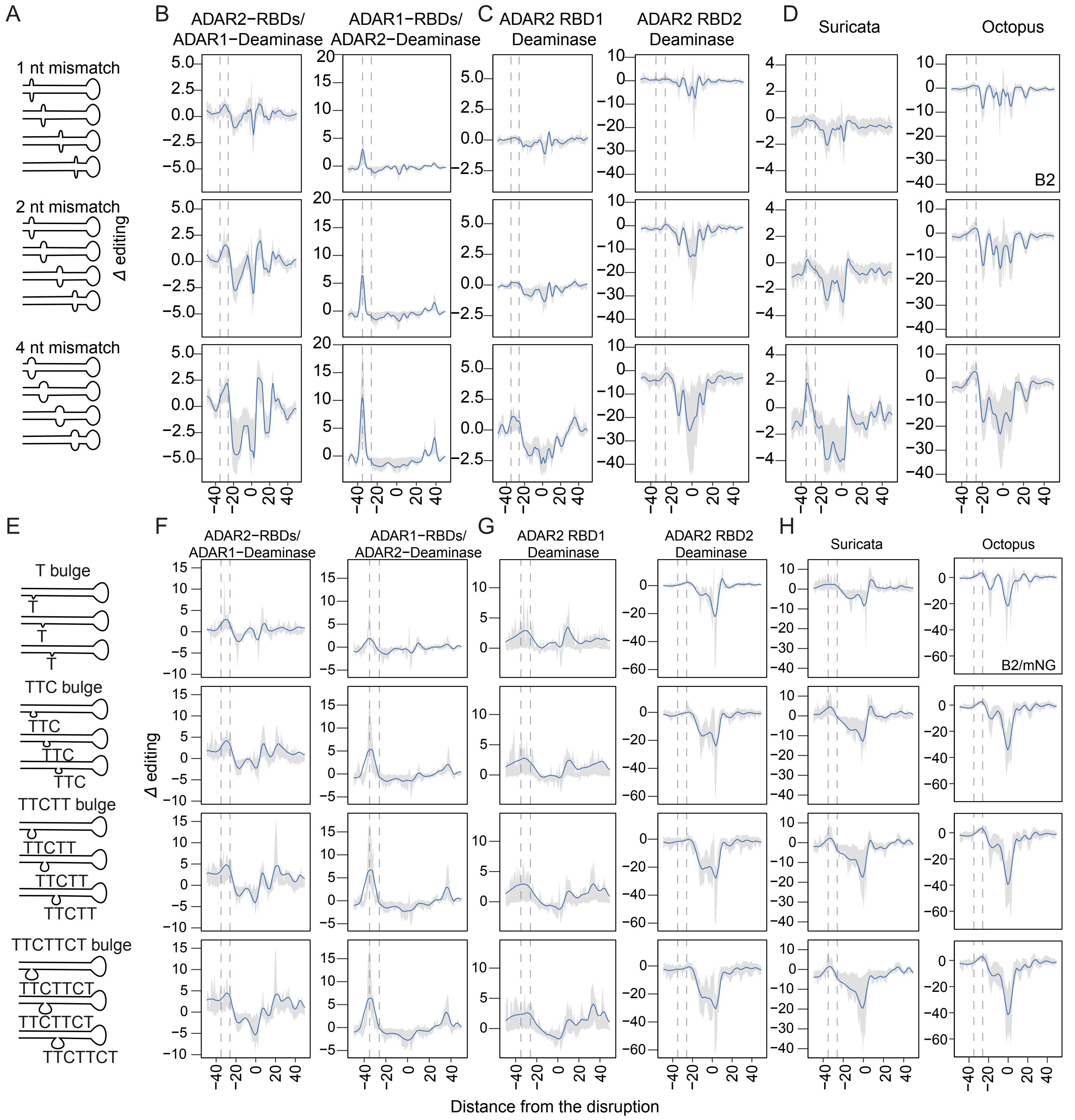

Figure S5. Editing is induced at ADAR-specific intervals upstream from structural disruptions. A) Graphical scheme of subsets of B2 constructs carrying 1, 2 or 4 bp mismatches along the stem structures. B) 'ADAR2-RBDs_ADAR1-deaminase' and 'ADAR1-RBDs_ADAR2-deaminase'-mediated editing offsets based on subsets of 1-, 2- and 4-nucleotide mismatch running throughout the mNG and B2 sequences. Mismatches differentially located in each construct get centered at 0 on the x-axis. Δ editing level on the y-axis represents the change of editing level of an adenosine, normalized to the perfect double-stranded construct. Fitted curves depict LOESS fit of Δ editing with a span of 0.05. The shaded region spans the 25th Percentile and 75th percentile values of Δ editing per distance. Only adenosine positions, which have greater than 1% in editing, on the perfect double-stranded construct were included in the analysis. Vertical dashed lines are placed at -35 and -26. C) 'ADAR2 RBD1 deaminase' and 'ADAR2 RBD2 deaminase'-mediated editing offsets based on subsets of 1-, 2- and 4-nucleotide mismatch running throughout the mNG and B2 sequences. Data is shown as Figure S5B. D) 'Suricata'- and 'Octopus' ADAR-mediated editing offsets based on subsets of 1-, 2- and 4-nucleotide mismatch running throughout the mNG and B2 sequences. Data is shown as Figure S5B. E) Graphical scheme of subsets of B2 constructs carrying T, TTC, TTCTT, and TTCTTCT bulges along the stem structures. F) 'ADAR2-RBDs_ADAR1-deaminase' and 'ADAR1-RBDs_ADAR2-deaminase'-mediated editing offsets based on subsets of T, TTC, TTCTT and TTCTTCT bulge running throughout the B2 sequences. Data is shown as the LOESS fit curve (blue-colored) of Δ editing with a span of 0.11. G) 'ADAR2 RBD1 deaminase' and 'ADAR2 RBD2 deaminase'-mediated editing offsets based on subsets of T, TTC, TTCTT and TTCTTCT bulge running throughout the B2 sequences. Data is shown as in the Figure S5F. H) 'Suricata' and 'Octopus' ADAR-mediated editing offsets based on subsets of T, TTC, TTCTT and TTCTTCT bulge running throughout the B2 sequences. Data is shown as in the Figure S5F.

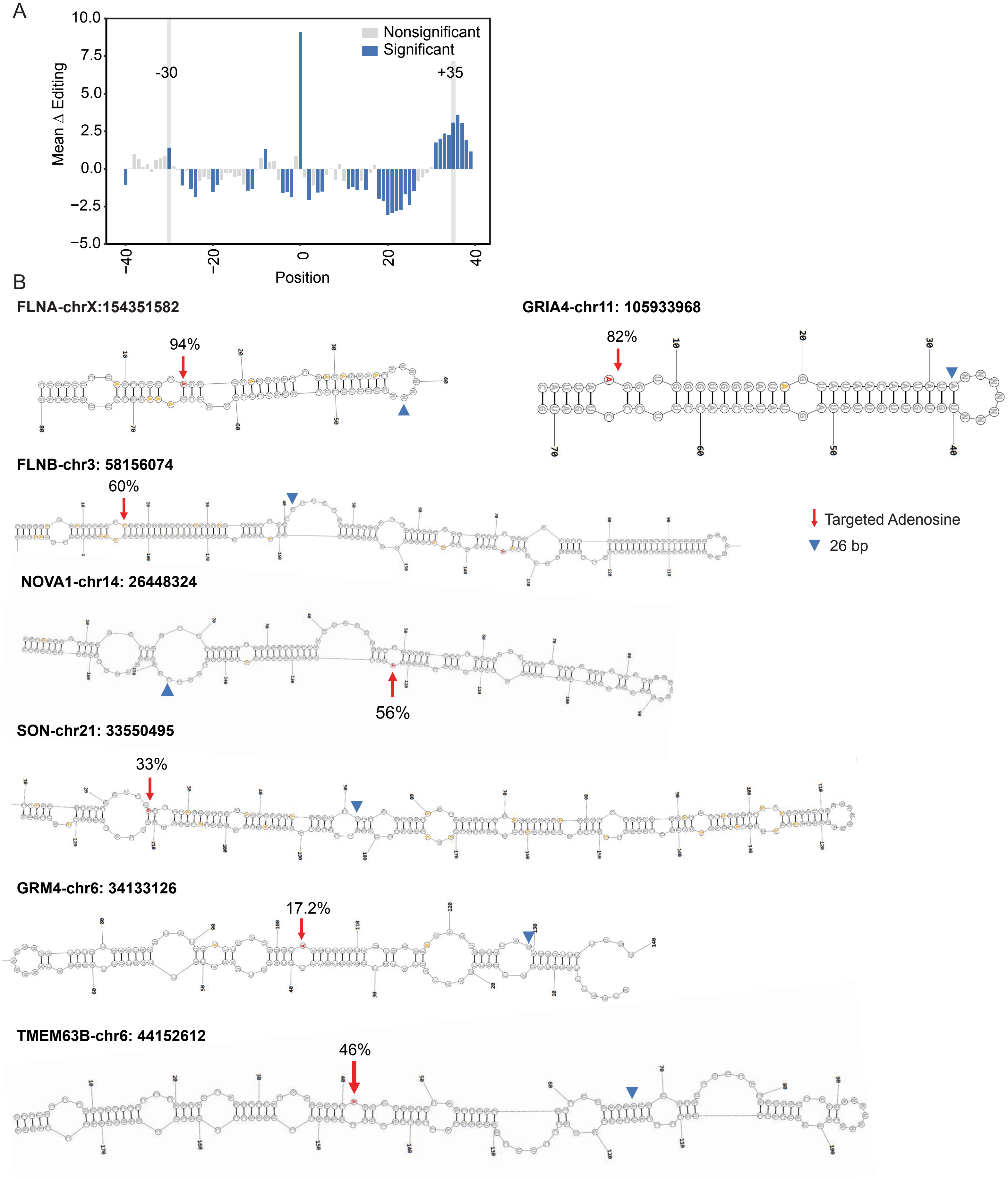

Figure S6. Editing is induced at ADAR-specific intervals upstream from structural disruptions on endogenous transcripts.
A) Meta-analysis comparing the difference in editing levels at a central position (position 0) among groups of transcripts harboring a structurally-open vs closed position. For this analysis, each relative position within a window of 40 nt centered around the edited site was considered separately, and all edited sites were binned into one of two groups (open or closed) based on the predicted structure at that position. The mean Δ editing level on the y-axis represents the change of mean editing level of an adenosine in open vs closed structures. Gray-highlighted vertical bars are placed at -30 and +35. Data is shown as the mean with n=9125 samples considered in total. Statistical comparisons were assessed using a t-test, and the significance ($p<0.05$) is visually represented through color coding. B) Analysis of adenosine editing within predicted secondary structures of known ADAR2-specific endogenous targets. The structures of FLNA, FLNB, GRM4, NOVA1, GRIA4, SON and TMEM63B transcripts were examined, highlighting the highly edited target (indicated by a red arrow), and the position 26 nt downstream of it (blue triangle). Editing levels of the target adenosine ranged from 0 (no A deamination) to 100% (complete A deamination).

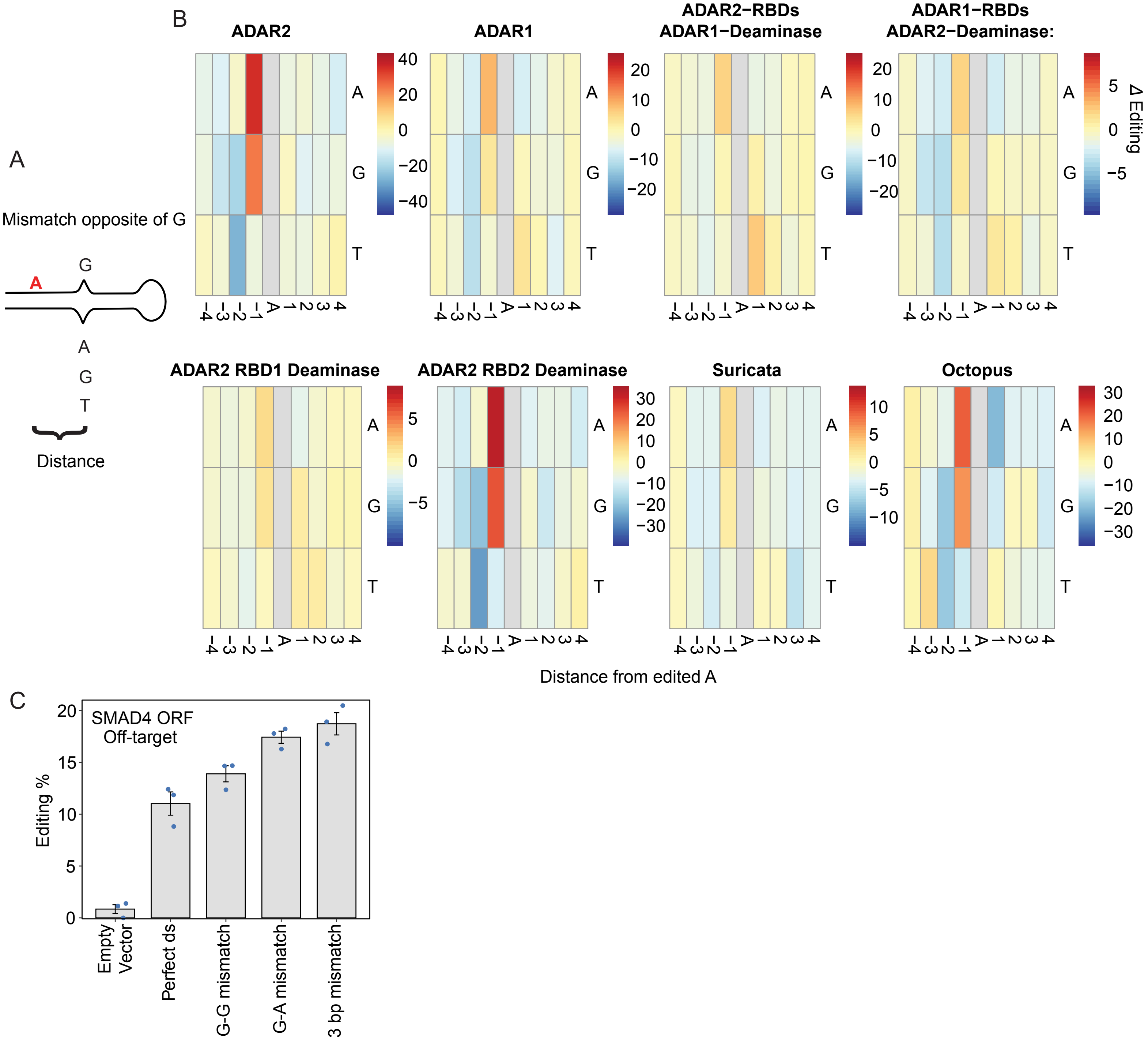

Figure S7. Effect of mismatching nearby bases on the opposite strand among ADARs. A) Graphical scheme of constructs harboring A, G, or T opposite to G. B) On the heatmaps, the x-axis shows the distance of the disruptions to the A site while the Y-axis shows to which base a G is mismatched. C) Quantification results showing the editing levels on off-targeted adenosine of the SMAD4 transcript in ADAR2-expressing cells. Data is shown as the mean ± s.e.m.(standard error of the mean) with n=3 independent experiments.
