# Supplementary material for: Dissecting the basis for differential substrate specificity of ADAR1 and ADAR2
Source: Nat Commun. 2023 Dec 11;14:8212. doi: 10.1038/s41467-023-43633-0 (PMC10713624; doi:10.1038/s41467-023-43633-0)
Supplement: Supplementary file 3 — Supplementary Data 1 [file 41467_2023_43633_MOESM3_ESM.pdf]

Supplementary Data 1. Primers and oligos

ID	Comment	Sequence
AC_mis_3nuc_26bp_SMAD4_UTR_151bp	SMAD4 targeting oligo, with 3 mismatched bases opposite of +26 from the targeted adenosine (gene block)	CAATCCGCCCTCACTACAACCGGAAATTTCCCGGGCCTTAAGCTAGCATGTTTTAAAAAAGGCTAGTGAGCTGGACGGCGACGTAAAGGTACCGTAGAATGATATACATATCGATGTTCTGCAGATAAAAGGCTCTAAGACCAATTAAAGGGCTAAATTTTCTAGCACCGGAATGATTCCTTCATACATAACAGGTGAAGAATTAATAAGAATGTGTTTCTCCTAATCTTCAAGCTCTGAGCCATTTTTTGGTACCGAACGCTGACGTCATCAACCATGATCTCTTTCGGCCGCGCATGCTGCATATAGTGTCATAGATCATCTACTCTGGCGTCGATGAGGGA
A_C_SMAD4_UTR_151bp	SMAD4 targeting positive control oligo (gene block)	CAATCCGCCCTCACTACAACCGGAAATTTCCCGGGCCTTAAGCTAGCATGTTTTAAAAAAGGCTAGTGAGCTGGACGGCGACGTAAAGGTACCGTAGAATGATATACATATCGATGTTCTGCAGATAAAAGGCTCTAAGACGTTTTAAAGGGCTAAATTTTCTAGCACCGGAATGATTCCTTCATACATAACAGGTGAAGAATTAATAAGAATGTGTTTCTCCTAATCTTCAAGCTCTGAGCCATTTTTTGGTACCGAACGCTGACGTCATCAACCATGATCTCTTTCGGCCGCGCATGCTGCATATAGTGTCATAGATCATCTACTCTGGCGTCGATGAGGGA
A_C_mis_3nuc_26bp_151bp_PPIB_UTR	PPIB UTR targeting oligo, with 3 mismatched bases opposite of +26 from the targeted adenosine (gene block)	CAATCCGCCCTCACTACAACCGGAAATTTCCCGGGCCTTAAGCTAGCATGTTTTAAAAAAGGCTAGTGAGCTGGACGGCGACGTAAAGGTACCGAATGTGAGGGGAGTGGGTCCGCTCCACCAGATGCCAGCACCGGGGCCTCAGCAGCTCAGAGCCCTGTGGCGGACCACAGGGCCTGCACAGACGGTCACTCAAAGAAAGATGTCCCTGTGCCCTACTCCTTGGCGATGGCAAAGGGCTTCTTTTTTTGGTACCGAACGCTGACGTCATCAACCATGATCTCTTTCGGCCGCGCATGCTGCATATAGTGTCATAGATCATCTACTCTGGCGTCGATGAGGGA
A_C_mis_151bp_PPIB_UTR	PPIB UTR targeting positive control oligo (gene block)	CAATCCGCCCTCACTACAACCGGAAATTTCCCGGGCCTTAAGCTAGCATGTTTTAAAAAAGGCTAGTGAGCTGGACGGCGACGTAAAGGTACCGAATGTGAGGGGAGTGGGTCCGCTCCACCAGATGCCAGCACCGGGGCCAGTGCAGCTCAGAGCCCTGTGGCGGACCACAGGGCCTGCACAGACGGTCACTCAAAGAAAGATGTCCCTGTGCCCTACTCCTTGGCGATGGCAAAGGGCTTCTTTTTTTGGTACCGAACGCTGACGTCATCAACCATGATCTCTTTCGGCCGCGCATGCTGCATATAGTGTCATAGATCATCTACTCTGGCGTCGATGAGGGA

A_Cmis_3_nuc_26bp_151bp_PPIB_ORF	PPIB ORF targeting oligo, with 3 mismatched bases opposite of +26 from the targeted adenosine (gene block)	CAATCCGCCCTCACTACAACCGGAAATTTCCCGGGCCTTAAGCTAGCATGTTTTAAAAAAGGCTAGTGAGCTGGACGGCGACGTAAAGGTACCGATCACATCCTTCAGGGGTTTATCCCGGCTGTCTGTCTTGGTGCTCTCGTGCTTCCGCACCACCTCCATGCCCTCCAGAACTTTGCCAAACACCACATGCTTGCCATCTAGCCAGGCTGTCTTGACTGTCGTGATGAAGAACTGGGAGCCGTTTTTTGGTACCGAACGCTGACGTCATCAACCATGATCTCTTTCGGCCGCGCATGCTGCATATAGTGTCATAGATCATCTACTCTGGCGTCGATGAGGGA
A_C_mis_151bp_PPIB_ORF	PPIB ORF targeting positive control oligo (gene block)	CAATCCGCCCTCACTACAACCGGAAATTTCCCGGGCCTTAAGCTAGCATGTTTTAAAAAAGGCTAGTGAGCTGGACGGCGACGTAAAGGTACCGATCACATCCTTCAGGGGTTTATCCCGGCTGTCTGTCTTGGTGCTCTCCACCTTCCGCACCACCTCCATGCCCTCCAGAACTTTGCCAAACACCACATGCTTGCCATCTAGCCAGGCTGTCTTGACTGTCGTGATGAAGAACTGGGAGCCGTTTTTTGGTACCGAACGCTGACGTCATCAACCATGATCTCTTTCGGCCGCGCATGCTGCATATAGTGTCATAGATCATCTACTCTGGCGTCGATGAGGGA
STAT1_A_C_mismatch_151bp	STAT1 targeting positive control oligo (gene block)	CAATCCGCCCTCACTACAACCGATTTCCCGGGCCTTAAGCTAGCATGTTTTAAAAAAGGCTAGTGAGCTGGACGGCGACGTAAAGGTACCATGGGGAGCAGGTTGTCTGTGGTCTGAAGTCTAGAAGGGTGAACTTCAGACACAGAAATCAACTCAGTCTTGATACATCCAGTTCCTTTAGGGCCATCAAGTTCCATTGGCTCTGGTGCTTCCTTTGGCCTGGAGTAATACTTTCCAAAGGTTTTTTGGTACCGAACGCTGACGTCATCAACCATGATCTCTTTCGGCCGCGCATGCTGCATATAGTGTCATAGATCATACTCTACTCTGGCGTCGATGAGGGA
STAT1_A_C_mis_3nucmis_27bp_151bp	STAT1 targeting oligo, with 3 mismatched bases opposite of +26 from the targeted adenosine (gene block)	CAATCCGCCCTCACTACAACCGATTTCCCGGGCCTTAAGCTAGCATGTTTTAAAAAAGGCTAGTGAGCTGGACGGCGACGTAAAGGTACCATGGGGAGCAGGTTGTCTGTGGTCTGAAGTCTAGAAGGGTGAACTTCTCTCACAGAAATCAACTCAGTCTTGATACATCCAGTTCCTTTAGGGCCATCAAGTTCCATTGGCTCTGGTGCTTCCTTTGGCCTGGAGTAATACTTTCCAAAGGTTTTTTGGTACCGAACGCTGACGTCATCAACCATGATCTCTTTCGGCCGCGCATGCTGCATATAGTGTCATAGATCATACTCTACTCTGGCGTCGATGAGGGA
RE_A_C_mis_151_GAPDH	GAPDH targeting positive control oligo (gene block)	ATTTCCCGGGCCTTAAGCTAGCATGTTTTAAAAAAGGCTAGTGAGCTGGACGGCGACGTAAAGGTACCGTTGAGCACAGGGTACTTTATTGATGGTACATGACAAGGTGCGGCTCCCTAGGCCCCTCCCCTCTTCAAGGGGTCCACATGGCAACTGTGAGGAGGGGAGATTCAGTGTGGTGGGGGACTGAGTGTGGCAGGGACTCCCCAGCAGTGAGGGTTTTTTGGTACCGAACGCTGACGTCATCAACCATGATCTCTTTCGGCCGCGCATGCTGCATATAGTGTCATAGATCATACT

RE_ACmis_151_GAPDH_27bp_3nuc_mis	GAPDH targeting oligo, with 3 mismatched bases opposite of +27 from the targeted adenosine (gene block)	ATTTCCCGGGCCTTAAGCTAGCATGTTTTAAAAAAGGCTAGTGAGCTGGACGGCGACGTAAAGGTACCGTTGAGCACAGGGTACTTTATTGATGGTACATGACAAGGTGCGGCTCGGAAGGCCCCTCCCCTCTTCAAGGGGTCCACATGGCAACTGTGAGGAGGGGAGATTCAGTGTGGTGGGGGACTGAGTGTGGCAGGGACTCCCCAGCAGTGAGGGTTTTTTGGTACCGAACGCTGACGTCATCAACCATGATCTCTTTCGGCCGCGCATGCTGCATATAGTGTCATAGATCATACT
SMAD4_perfect_ds_GA_context_151bp	SMAD4 targeting perfect double-stranded oligo (gene block)	CAATCCGCCCTCACTACAACCGGAAATTTCCCGGGCCTTAAGCTAGCATGTTTTAAAAAAGGCTAGTGAGCTGGACGGCGACGTAAAGGTACCAAGGTTAAGGGCCCCAACGGTAAAAGACCTCAGTCTAAAGGTTGTGGGTCTGCAATCGGCATGGTATGAAGTACTTCGTCTAGGAGCTGGAGGGCCCGGTGTAAGTGAATTTCAATCCAGCAAGGTGTTTCTTTGATGCTCTGTCTTGGGTTTTTTTGGTACCGAACGCTGACGTCATCAACCATGATCTCTTTCGGCCGCGCATGCTGCATATAGTGTCATAGATCATCTACTCTGGCGTCGATGAGGGA
SMAD4_A_mismatch_GA_context_151bp	SMAD4-targeting oligo, with A mismatched bases opposite of -1 from the targeted adenosine (gene block)	CAATCCGCCCTCACTACAACCGGAAATTTCCCGGGCCTTAAGCTAGCATGTTTTAAAAAAGGCTAGTGAGCTGGACGGCGACGTAAAGGTACCAAGGTTAAGGGCCCCAACGGTAAAAGACCTCAGTCTAAAGGTTGTGGGTCTGCAATCGGCATGGTATGAAGTACTTAGTCTAGGAGCTGGAGGGCCCGGTGTAAGTGAATTTCAATCCAGCAAGGTGTTTCTTTGATGCTCTGTCTTGGGTTTTTTTGGTACCGAACGCTGACGTCATCAACCATGATCTCTTTCGGCCGCGCATGCTGCATATAGTGTCATAGATCATCTACTCTGGCGTCGATGAGGGA
SMAD4_G_mismatch_GA_context_151bp	SMAD4-targeting oligo, with G mismatched bases opposite of -1 from the targeted adenosine (gene block)	CAATCCGCCCTCACTACAACCGGAAATTTCCCGGGCCTTAAGCTAGCATGTTTTAAAAAAGGCTAGTGAGCTGGACGGCGACGTAAAGGTACCAAGGTTAAGGGCCCCAACGGTAAAAGACCTCAGTCTAAAGGTTGTGGGTCTGCAATCGGCATGGTATGAAGTACTTGGTCTAGGAGCTGGAGGGCCCGGTGTAAGTGAATTTCAATCCAGCAAGGTGTTTCTTTGATGCTCTGTCTTGGGTTTTTTTGGTACCGAACGCTGACGTCATCAACCATGATCTCTTTCGGCCGCGCATGCTGCATATAGTGTCATAGATCATCTACTCTGGCGTCGATGAGGGA
SMAD4_3_bp_mismatch_GA_context_151bp	SMAD4-targeting oligo, with 3 mismatched bases opposite to the targeted adenosine (gene block)	CAATCCGCCCTCACTACAACCGGAAATTTCCCGGGCCTTAAGCTAGCATGTTTTAAAAAAGGCTAGTGAGCTGGACGGCGACGTAAAGGTACCAAGGTTAAGGGCCCCAACGGTAAAAGACCTCAGTCTAAAGGTTGTGGGTCTGCAATCGGCATGGTATGAAGTACAAGGTCTAGGAGCTGGAGGGCCCGGTGTAAGTGAATTTCAATCCAGCAAGGTGTTTCTTTGATGCTCTGTCTTGGGTTTTTTTGGTACCGAACGCTGACGTCATCAACCATGATCTCTTTCGGCCGCGCATGCTGCATATAGTGTCATAGATCATCTACTCTGGCGTCGATGAGGGA
Geneblock PCR F	PCR primer to amplify gene blocks	gagctggacggcgacgtaaa

Geneblock PCR R	PCR primer to amplify gene blocks	Ggttgatgacgtcagcgttc
RW_EPB104_U6_colony	PCR primer to amplify and linearize flanking sequences of EPB104 plasmid and arRNA	GAGCGAGGAAGCGGAGAGC
FW_EPB104_U6_Colony	PCR primer to amplify and linearize flanking sequences of EPB104 plasmid and arRNA	CGTGTCTTGTCCAGAGCTCGATATC
SMAD4_ORF_F	PCR primer to amplify SMAD4 targeted mRNA	GTGTTGATGACCTTCGTCGCTTATG
SMAD4_ORF_R	PCR primer to amplify SMAD4 targeted mRNA	CAGGATTGTATTTTGTAGTCCACCATCC
FW_STAT1_gRNA_MZ	PCR primer to amplify STAT1 targeted mRNA	cgaacctgacttccatgcgg
RW_STAT1.1_gRNA_MZ	PCR primer to amplify STAT1 targeted mRNA	GAGAAGGAAAACTGTCGCCAGAG
FW_SMAD4_UTR_gRNA_MZ2	PCR primer to amplify SMAD4 UTR targeted mRNA	TTCCTCTTCAGAACTTGTCAGGCA

RW_SMAD4_UTR_gRNA_MZ2	PCR primer to amplify SMAD4 UTR targeted mRNA	CTCACCCACATGGCACATTATTTTTG
FW_amp_GAPDH_MZ	PCR primer to amplify GAPDH targeted mRNA	TCTTTCCCTACACGACGCTCTTCCGATCTCCCAGCAAGAGCACAAGAGGAAG
RW_amp_GAPDH_MZ	PCR primer to amplify GAPDH targeted mRNA	TGGAGTTCAGACGTGTGCTCTTCCGATCTGTACATGACAAGGTGCGGCTC
FW_gRNA_PPIB_MZ	targeted mRNA	GATGGCACAGGAGGAAAGAGCATC
RW_gRNA_PPIB_MZ	targeted mRNA	GTTACAAAAGTGAGTCCATGGGCC
SMAD4_R_GA_context	PCR primer to amplify SMAD4 targeted mRNA (GA context)	CAGGATTGTATTTTGTAGTCCACCATCC
SMAD4_F_GA_context	PCR primer to amplify SMAD4 targeted mRNA (GA context)	CTGTCAGCTGCTGCTGGAATTG
RW_HindIII_ADAR1	sequences include restriction sites for cloning hADAR1	ACCTAATTATAACGTTCTAATGGTGATGGTGATGGTGTACTGG
FW_XBAI_ADAR1_Flag	sequences include restriction sites for cloning hADAR1	GAATCGAATGTCTAGAATGGACTACAAGGACGACGATGAC
MZpcDNA31_ECORI_RW	sequences include restriction sites for cloning hADAR2	GATGATCTGTGAATTCTCAGACTAGGGGCGTGAGTGAGAAC

MZ_pcDNA31_Xbai_FW	sequences include restriction sites for cloning hADAR2	CAGCATACGATCTAGAATGGACTACAAGGACGACGATGAC
MZ_pcDNA3.1_mutant_hADAR2_xbal_FW	sequences include restriction sites for cloning mutant ADAR2	GATGATCTGTGAATTCTCAATGGTGATGGTGATGGTGGACTAGG
MZ_pcDNA3.1_mutant_hADAR2_xbal_RW	sequences include restriction sites for cloning mutant ADAR2	GCAGCATCGATCTAGAATGGACTACAAGGACGACGATGACAAAC
H3_INS_FW	sequences for ADAR1-ADAR2 hybrids for Gibson Assembly	GTCATGGCTTGCCACGGTGTCTCCCCAAGAACGCCCTG
H3_BB_RW	sequences for ADAR1-ADAR2 hybrids for Gibson Assembly	ATCAGGGCGTTCTTGGGGAGACACCGTGGCAAGCCATG
H3_BB_FW	sequences for ADAR1-ADAR2 hybrids for Gibson Assembly	CCGCCATTTTTAACTTGCACTTGGATACAGAGGTAACCCCAGTG
H3_INS_RW	sequences for ADAR1-ADAR2 hybrids for Gibson Assembly	CCTGTCACTGGGGTTACCTCTGTATCCAAGTGCAAGTTAAAAATG
H4_INS_FW	sequences for ADAR1-ADAR2 hybrids for Gibson Assembly	GGAAAACACCAGGGCCCGTCCGGTGTTCACCCTACAAG
H4_BB_RW	sequences for ADAR1-ADAR2 hybrids for Gibson Assembly	CAGTTTCTTGTAGGGTGAACACCGGACGGGCCCTGGTGTTTTC

H4_BB_FW	sequences for ADAR1-ADAR2 hybrids for Gibson Assembly	GAAGGCAGAACGCATGGGTTTCTTGCACTTGGATCAGAC
H4_INS_RW	sequences for ADAR1-ADAR2 hybrids for Gibson Assembly	GGCGTCTGATCCAAGTGCAAGAAACCCATGCGTTCTG
B2 PCR F	PCR primer for targeted amplification and NGS of B2 oligo pool	AATGATACGGCGACCACCGAGATCTACACTCTTTCC CTACACGACGCTCTTCCGATCT GGACGAGCTGTACAAGTAAACTAGT
mNG PCR F	PCR primer for targeted amplification and NGS of mNG oligo pool	AATGATACGGCGACCACCGAGATCTACACTCTTTCC CTACACGACGCTCTTCCGATCT ACCACCTACACCTTTGCCA
RT	RT primer for targeted amplification and NGS of B2 and mNG oligo pools	AGACGTGTGCTCTTCCGATCT TGGGTTGAGGGTAGTGAG
RT_variable_half	RT primer for targeted amplification and NGS of B2 second (variable) strand	AGACGTGTGCTCTTCCGATCT gatatcgaatGGCGCGCC
B2_F_variable	PCR primer for targeted amplification and NGS of B2 second (variable) strand	AATGATACGGCGACCACCGAGATCTACACTCTTTCCCTACACGACGCTCTTCCGATCT CAAAACCCCTAAAACCTTCGAA
