# Supplementary material for: Dissecting the basis for differential substrate specificity of ADAR1 and ADAR2
Source: Nat Commun. 2023 Dec 11;14:8212. doi: 10.1038/s41467-023-43633-0 (PMC10713624; doi:10.1038/s41467-023-43633-0)
Supplement: Supplementary file 5 — Description of Additional Supplementary Files [file 41467_2023_43633_MOESM5_ESM.pdf]

Description of Additional Supplementary Files

File Name: Supplementary Data 1
Description: Primers and Oligos. This supplementary data file includes a comprehensive list of primers and oligonucleotides described in the experimental methods. It provides detailed information on the sequences, and specific applications of each primer or oligo.

File Name: Supplementary Data 2
Description: The R script for analyzing NGS sequencing data from the B2 and mNG oligo libraries transfected along with ADAR-specific plasmid in ADAR1 KO-HEK293T cell lines
