# Supplementary material for: Dissecting the basis for differential substrate specificity of ADAR1 and ADAR2
Source: Nat Commun. 2023 Dec 11;14:8212. doi: 10.1038/s41467-023-43633-0 (PMC10713624; doi:10.1038/s41467-023-43633-0)
Supplement: Supplementary file 6 — Reporting Summary [file 41467_2023_43633_MOESM6_ESM.pdf]

nature portfolio

Corresponding author(s): Prof. Schraga Schwartz

Last updated by author(s): Nov 6, 2023

# Reporting Summary

Nature Portfolio wishes to improve the reproducibility of the work that we publish. This form provides structure for consistency and transparency in reporting. For further information on Nature Portfolio policies, see our Editorial Policies and the Editorial Policy Checklist.

## Statistics

For all statistical analyses, confirm that the following items are present in the figure legend, table legend, main text, or Methods section.

| n/a | Confirmed | |
|---|---|---|
| ☐ | ☒ | The exact sample size (*n*) for each experimental group/condition, given as a discrete number and unit of measurement |
| ☒ | ☐ | A statement on whether measurements were taken from distinct samples or whether the same sample was measured repeatedly |
| ☐ | ☒ | The statistical test(s) used AND whether they are one- or two-sided<br>*Only common tests should be described solely by name; describe more complex techniques in the Methods section.* |
| ☒ | ☐ | A description of all covariates tested |
| ☒ | ☐ | A description of any assumptions or corrections, such as tests of normality and adjustment for multiple comparisons |
| ☐ | ☒ | A full description of the statistical parameters including central tendency (e.g. means) or other basic estimates (e.g. regression coefficient) AND variation (e.g. standard deviation) or associated estimates of uncertainty (e.g. confidence intervals) |
| ☐ | ☒ | For null hypothesis testing, the test statistic (e.g. *F*, *t*, *r*) with confidence intervals, effect sizes, degrees of freedom and *P* value noted<br>*Give P values as exact values whenever suitable.* |
| ☒ | ☐ | For Bayesian analysis, information on the choice of priors and Markov chain Monte Carlo settings |
| ☒ | ☐ | For hierarchical and complex designs, identification of the appropriate level for tests and full reporting of outcomes |
| ☐ | ☒ | Estimates of effect sizes (e.g. Cohen's *d*, Pearson's *r*), indicating how they were calculated |

*Our web collection on statistics for biologists contains articles on many of the points above.*

## Software and code

Policy information about availability of computer code

| | |
|---|---|
| Data collection | Illumina NovaSeq platforms were employed for data collection. |
| Data analysis | A custom R (v 4.0.0) script was employed to evaluate the Fastq files and to quantify the single-base resolution A-to-I editing per construct on B2 and mNG library as described in the Methods subsection Data analysis of NGS data. Sanger-sequencing-based editing quantification was performed using the EditR tool (Kluesner, M. G. et al., 2018), and MultiEditR (Kluesner, M. G. et al., 2021). For analyzing the A-to-I deamination on GAPDH, all Fastq FILES obtained from the amplicon sequencing libraries were analyzed and evaluated using a custom R script as described in Methods subsection GAPDH amplicon library preparation and analysis of sequencing data. The R script for analyzing NGS sequencing data from the B2 and mNG oligo libraries is accessible as a Supplementary Data 2. |

For manuscripts utilizing custom algorithms or software that are central to the research but not yet described in published literature, software must be made available to editors and reviewers. We strongly encourage code deposition in a community repository (e.g. GitHub). See the Nature Portfolio guidelines for submitting code & software for further information.

## Data

Policy information about availability of data

All manuscripts must include a data availability statement. This statement should provide the following information, where applicable:

- Accession codes, unique identifiers, or web links for publicly available datasets
- A description of any restrictions on data availability
- For clinical datasets or third party data, please ensure that the statement adheres to our policy

All datasets generated for this manuscript have been deposited in the NCBI BioProject database under the ID PRJNA943413.
The datasets pertaining to the expression of B2 and mNG constructs in WT HEK293T cells (Figure 1) were obtained from Uzonyi et al. 2021.
For the analysis of ADAR1 and ADAR2 endogenous targets, data were retrieved from the GTEX database. The R script for analyzing NGS sequencing data from the B2 and mNG oligo libraries is accessible as a Supplementary Data 2.

## Human research participants

Policy information about studies involving human research participants and Sex and Gender in Research.

| | |
|---|---|
| Reporting on sex and gender | Not applicable |
| Population characteristics | Not applicable |
| Recruitment | Not applicable |
| Ethics oversight | Not applicable |

Note that full information on the approval of the study protocol must also be provided in the manuscript.

# Field-specific reporting

Please select the one below that is the best fit for your research. If you are not sure, read the appropriate sections before making your selection.

☒ Life sciences ☐ Behavioural & social sciences ☐ Ecological, evolutionary & environmental sciences

For a reference copy of the document with all sections, see nature.com/documents/nr-reporting-summary-flat.pdf

# Life sciences study design

All studies must disclose on these points even when the disclosure is negative.

| | |
|---|---|
| Sample size | Statistical methods were not employed in determining the sample size; instead, it was selected based on practical considerations. The B2 and mNG construct libraries were independently transfected in technical duplicates. The sample size for the recruitment of arRNAs for target RNA editing was intentionally set to attain a sample size of three, as consistently mentioned in both the manuscript and the legend of the relevant figure. |
| Data exclusions | The editing thresholds on Adenosine positions in the editing-induction plots among ADARs for the oligo-library data analysis were set, as described throughout the manuscript |
| Replication | The B2 and mNG construct libraries were transfected independently in technical duplicates, and the recruitment of arRNAs for target RNA editing was performed in technical replicates. Importantly, it is worth mentioning that all attempts at replication yielded successful results. |
| Randomization | In such experimental setups, randomization was not suitable because the grouping and treatment assignments were predetermined based on the unique characteristics and objectives of each experiment. Therefore, randomization was not a relevant or appropriate consideration for this particular research. |
| Blinding | Blinding was not applicable to the study due to the nature of the experiments conducted. Blinding was not feasible as it would not have affected the execution or interpretation of these specific and distinct experimental procedures, making it unnecessary for this research. |

# Reporting for specific materials, systems and methods

We require information from authors about some types of materials, experimental systems and methods used in many studies. Here, indicate whether each material, system or method listed is relevant to your study. If you are not sure if a list item applies to your research, read the appropriate section before selecting a response.

## Materials & experimental systems

| n/a | Involved in the study | |
|---|---|---|
| ☐ | ☒ | Antibodies |
| ☐ | ☒ | Eukaryotic cell lines |
| ☒ | ☐ | Palaeontology and archaeology |
| ☒ | ☐ | Animals and other organisms |
| ☒ | ☐ | Clinical data |
| ☒ | ☐ | Dual use research of concern |

## Methods

| n/a | Involved in the study | |
|---|---|---|
| ☒ | ☐ | ChIP-seq |
| ☒ | ☐ | Flow cytometry |
| ☒ | ☐ | MRI-based neuroimaging |

# Antibodies

Antibodies used

Antibodies used were anti-mouse FLAG (1:2000, Sigma, F3165), anti-goat Actin HRP (1:5000, Jackson, 805-035-180) and anti-mouse HRP (1:5000, abcam, ab97040).

Validation

We ensured the validation of all commercially obtained antibodies by cross-referencing the manufacturers' validation statements and consulting previously published studies that have effectively employed these antibodies.

# Eukaryotic cell lines

Policy information about cell lines and Sex and Gender in Research

Cell line source(s)

Human ADAR (ADAR1) knockout HEK-293T cell line was obtained from Stetson's Lab (described in Pestal et al. 2015)

Authentication

ADAR1-KO HEK293T cell line was not authenticated

Mycoplasma contamination

The cell lines underwent mycoplasma testing using the MycoBlue Mycoplasma Detector (Vazyme Catalog # D101), and the results confirmed the absence of mycoplasma contamination in the Human ADAR (ADAR1) knockout HEK-293T cell line.

Commonly misidentified lines
(See ICLAC register)

No commonly misidentified lines were employed.
